# Incisors’ bone height and inclination changes after orthodontic treatment with a self-ligating passive system

**DOI:** 10.4317/jced.60669

**Published:** 2023-08-01

**Authors:** Célia-Regina-Maio Pinzan-Vercelino, Karina-Maria-Salvatore Freitas, Monique Secco, Arnaldo Pinzan, Paula Cotrin, Fabricio-Pinelli Valarelli, Guilherme Janson, Marcos-Roberto Freitas

**Affiliations:** 1 Centro Universitário Uningá, Departamento de Odontologia (Maringá/PR, Brazil); 2 Department of Orthodontics, Bauru Dental School, University of São Paulo, Bauru, São Paulo, Brazil

## Abstract

**Background:**

This study aimed to evaluate changes in the alveolar buccal bone height of maxillary and mandibular incisors after orthodontic treatment with a self-ligating passive system and to assess the correlation between bone height and incisor inclination.

**Material and Methods:**

Pre (T1) and post-treatment (T2) cone-beam computed tomography images of patients treated with the Damon 3MX appliance system were measured to quantify the alveolar buccal bone height of the maxillary incisors. The incisor’s inclination was measured in digital models. Paired t-test was used to evaluate the changes between T1 and T2, and Pearson’s coefficient was used to test the correlation.

**Results:**

All teeth presented statistically significant alveolar buccal bone loss at T2. A statistically significant buccal inclination was observed only for the lower left lateral incisors. There was no correlation between bone height changes and incisor inclination.

**Conclusions:**

Orthodontic treatment with a self-ligating passive system showed changes in alveolar height, but these changes were not correlated with incisor inclination.

** Key words:**Passive self-ligating brackets, orthodontics, corrective, treatment outcome, alveolar bone loss.

## Introduction

Crowding alleviation in conventional nonextraction orthodontic treatment is achieved by dental inclinations, with an increase in arch length, incisor proclination, and the transversal arch dimensions (1-6). These dental changes may occur irrespective of the bracket type used (self-ligating passive, self-ligating active, and conventional brackets) (3-8).

Self-ligation brackets are frequently used in orthodontic practice. Their popularity possibly results from effective marketing and advertising (9). The advantages claimed by the manufacturers are low friction, light forces, reduction in the number of extractions, less chair time, and greater appointment intervals. Different self-ligating systems are available, including the Damon system.

According to Damon (10), self-ligating brackets and broader archwires have some advantages over traditional conventional fixed appliances. One of the key benefits is that they can promote posterior expansion without simultaneous incisor proclination, because low friction occurs between the bracket and wire, which allows for light force to be applied to the teeth. This helps to reduce the pressure on the teeth, which can help minimize discomfort during treatment. Besides, the perioral muscles act as a “lip bumper,” minimizing the anterior movement of the incisors (10). To the best of our knowledge, no scientific evidence corroborates this statement.

Some studies have suggested that dental proclination, which is the forward movement of teeth away from the alveolar center in the direction of the buccal cortical plate, can increase the risk of alveolar bone defects and gingival recession (11,12). Therefore, orthodontists should carefully consider the amount of dental proclination required to achieve optimal alignment and occlusion, mainly in borderline nonextraction treatments.

Cone-beam computed tomography (CBCT) has been shown to be an accurate tool for dental and bone measurements. CBCT can provide detailed information about the position, size, and shape of teeth and bones, making it useful for orthodontic diagnosis and treatment planning (13-19). Several studies have investigated the accuracy of CBCT in dental and bone measurements and have found it to be highly precise and reliable (20).

Orthodontic treatment aims to achieve the best esthetic and occlusal results in the shortest possible time and with minimal dental and periodontal structure damage. There is a lack of evidence regarding the use of CBCT to assess the loss of vertical bone support in the lower incisors after treatment with Damon system self-ligating brackets. Previous CBCT studies evaluated only the mandibular arch (4,21,22) or after the alignment and leveling stage (23). Thus, our purpose was to evaluate changes in the marginal alveolar buccal bone height of maxillary and mandible incisors after orthodontic treatment with a self-ligating passive system and to assess the correlation between bone height and incisor inclination.

## Material and Methods

The local institutional review board approved this study (protocol #1.334.922).

The sample size calculation was based on an alpha significance level of 5% and a beta of 20% to achieve 80% of test power to detect a minimum difference of 2.65mm, with a standard deviation of 2.26 for the alveolar height (22). A sample size of 12 patients was determined.

The sample was retrospectively selected from the files of the Bauru Dental School – University of São Paulo according to the following inclusion criteria: patients with bilateral Angle Class I malocclusion at pretreatment; patients treated with the self-ligating passive system; nonextraction treatment in both arches; moderate maxillary and mandibular crowding (4–6 mm); patients aged between 12 and 17 years at pretreatment; all permanent teeth up to the second molars should be present; absence of dental anomalies of number; absence of impacted teeth; good periodontal health; no pre-existing orthodontic treatment; pre and posttreatment CBCT exams. Patients with severe asymmetry, poor periodontal health, congenital craniofacial deformities, and/or low-quality CBCT images were excluded.

The orthodontic treatment was carried out with 0.022-in Damon 3MX appliance system, standard torque (Ormco/A Company, San Diego, USA) in a graduate orthodontic clinic. The same experienced supervisor guided and accompanied all treatments. All patients were treated with the following archwire sequence: 0.014-in and 0.018 x 0.025-in copper-nickel-titanium and 0.019 x 0.025-in stainless steel archwire. No palatal arches, quad-helices, palatal expanders, lip bumpers, or distalizing appliances were used. The appliances were removed when Class I canine and molar relationship, good alignment, overjet, and overbite were achieved.

The sample comprised 192 CBCT images and 24 digital models of 12 patients (male: 10, female: 2). The mean initial and final ages were 14.9 years (SD = 1.16), and 17.73 years (SD = 1.11), respectively. The treatment time was 2.83 years (SD = 0.8). The maxillary and mandibular right and left central and lateral incisors were evaluated.

The CBCT images were acquired before orthodontic treatment (T1) and at the end of orthodontic treatment (T2) using an i-CAT Classic scanner (Imaging Sciences International, Hatfield, USA), applying the following parameters: 20-second scan time with a 16-cm (diameter) x 13-cm (height) field of view at a resolution of 0.3 mm voxels. The images were obtained at 120 kV(p) and 5mA. Raw data were reconstructed and exported as DICOM files and then were imported into E-vol DX (CDT Software, São Paulo, Brazil) for analysis.

**Figure 1:**
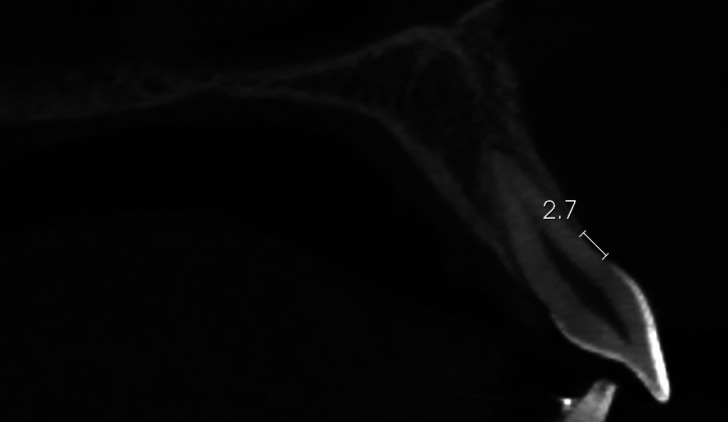
Measurement form CBCT: distance from the most apical portion of CEJ to the most coronal aspect of the buccal marginal bone crest, parallel to the long axis of the teeth.

The alveolar buccal bone height measurements were performed according to Garlock *et al*. (22). The CBCT image was oriented along the long axis of the tooth (bisecting the pulp and the canal) in the sagittal and coronal planes and bisecting the canal in a labiolingual direction in the axial plane at the same time. Once oriented, a sagittal cross-section of the incisor was produced. From this image, distances were made from the most apical portion of the CEJ to the most coronal aspect of the marginal bone crest (21,22). The measurements of the CBTC images were performed on the buccal surface parallel to the long axis of the teeth (13,16,22) (Fig. 1).

Pre- and posttreatment dental models were digitized using a 3D-scanner (model R-700; 3Shape, Copenhagen, Denmark), and the images were saved in .stl format. Angular measurements were made using a splicing function with the OrthoAnalyzer software (3Shape, Copenhagen, Denmark).

A single investigator conducted the measurements. The CBCT images and digital models from 38 teeth were randomly selected, and the same examiner re-measured the images 30 days later. The casual error was calculated according to Dahlberg’s formula (S2 = Ʃd2/2n), and the systematic error with the dependent t-test for *p* < .05.

The difference between T2 and T1 (T2-T1) was calculated to evaluate the bone and tooth changes. The normality of the data was checked with the Kolmogorov-Smirnov test, and the data presented normal distribution. A 1-sample t-test was used to evaluate the changes between the pre- and post-treatment measurements. The Pearson correlation coefficient analyzed the associations between bone and tooth variables. The level of significance was set at 5%, and all analyses were performed with SPSS software (version 20; IBM Corporation, Armonk, USA).

## Results

No systematic errors were detected for bone height measurements, and casual errors varied from 0.19mm to 0.38mm. For incisor inclination measurements, the range of casual errors varied from 0.3° to 0.7°, and only one variable showed a significant systematic error.

Changes in the alveolar buccal bone height of the maxillary and mandibular central and lateral right and left incisors were observed. The bone loss was statistically significant between the stages evaluated (Table 1).

**Table 1:**
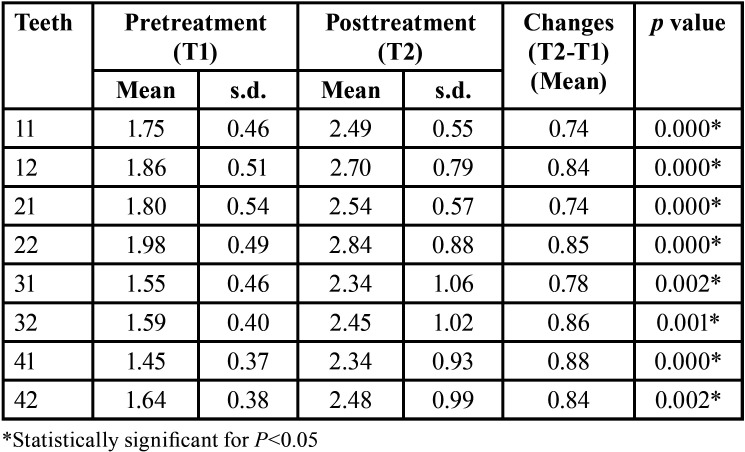
Descriptive statistics for alveolar buccal bone height and 1-sample t test p values to compare changes.

Different dental inclinations were observed between the incisors. After treatment, a statistically significant proclination was observed only for the mandibular left lateral incisor. Incisor proclination was observed for the maxillary central and lateral right incisors and mandibular central and lateral right incisors but without statistical significance. The maxillary central and lateral left incisors and mandibular left central incisors showed lingual inclination after treatment (Table 2), also without statistical significance.

**Table 2:**
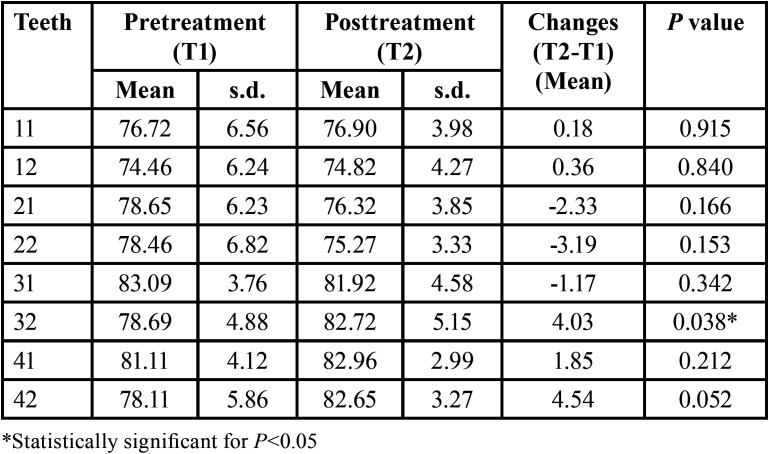
Descriptive statistics for incisor inclinations and 1-sample t test p values to compare changes.

Incisor inclination was not correlated with alveolar bone-height changes (Table 3).

**Table 3:**
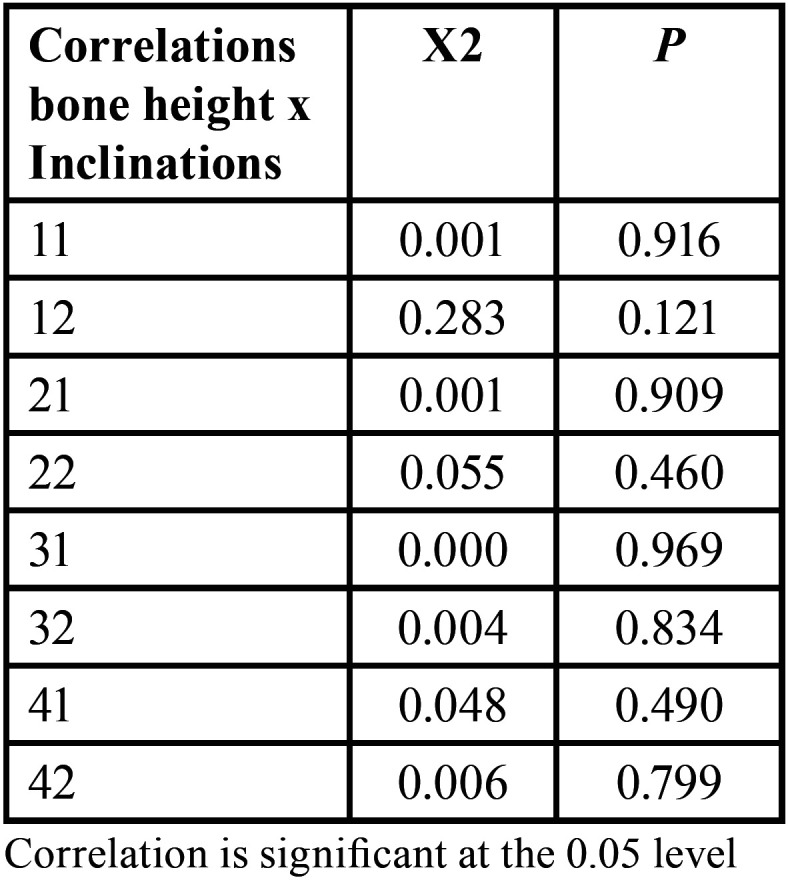
Correlations between bone and dental changes (Pearson).

## Discussion

Some studies claim that self-ligating brackets are a valuable tool in orthodontic treatment that can streamline clinical procedures, increase treatment efficiency, and reduce overall treatment time; however, there are scarce and contradictory results (4-7,24-28). The rationale behind our study is that the literature on bone changes using CBTC in maxillary and mandibular incisors at the end of orthodontic treatment with Damon self-ligating passive system is still sparse, so our aim was to accurately assess the bone changes that occur in the incisors after treatment with self-ligating.

There was a significant marginal bone loss for all the mandibular and maxillary incisors (Table 1). This finding agrees with previous studies that observed marginal bone loss in CBCT images after orthodontic treatment in cases with and without extractions (21-23,27). Garlock *et al*. (22) found an average of buccal vertical bone loss in mandibular right central incisors of 1.12 mm. Their result was very similar to our finding for the same tooth (0.88 mm). Morais *et al*. (23) also found that bone height increased significantly in the incisors (0.4mm for the maxillary and 0.3mm for the mandibular incisors) after alignment and leveling of teeth using Damon self-ligating brackets.

Our findings showed differences in inclinations between the incisors (Table 2). Cattaneo *et al*. (4) compared the central and lateral incisors and canines and observed great intra-arch variation in the inclination between adjacent teeth after orthodontic treatment. This result might be related to differences in initial crowding between teeth, inaccurate vertical bonding of the brackets, adjustment between the wire and bracket slots, and differences in tooth crown morphology (4,29).

There was no significant incisor proclination at posttreatment, except for the mandibular left lateral incisor (Table 2). Similar results were found in other studies (3,22). Vajaria *et al*. (3) observed statistically significant greater proclination in mandibular incisors. Still, maxillary incisors were not significantly proclinated after treatment with the Damon system. Garlock *et al*. (22) found statistically significant incisor proclination; however, the authors described that incisor-mandibular plane angle (IMPA) changes were highly variable, averaging 2.4°. Our results differed from those reported by Cattaneo *et al*. (4), who observed using CBTC significant mandibular incisor proclination. Morais *et al*. (23) found incisor buccal inclination after the alignment and leveling stage with Damon self-ligating brackets. Most likely, measurement protocol and methodology differences may explain this fact.

Although not statistically significant, the maxillary left central and lateral incisors and mandibular left central incisors showed lingual inclination. Our results may have occurred due to the tendency of the Damon system to increase arch dimensions at the alignment phase (7), as intercanine, interpremolar and intermolar width, creating some spaces that were closed with anterior tooth retroclination. We must consider that the Damon system’s philosophy advocates using broader archwires of copper-nickel-titanium and stainless steel. The Damon archwires have increased incisal curvature and posterior transverse distances, permitting great expansion. Moreover, these teeth showed greater proclination before treatment, which was likely corrected during treatment.

We did not observe a correlation between bone and dental changes, corroborating the findings of Garlock *et al*. (22). It is essential to highlight that we measured the incisor inclination without considering translation or vertical movements, which could affect vertical bone loss (22). These authors presumed that translation of the tooth caused bone loss, as they observed that on the surface where vertical bone recession occurred, thinning of the cortical bone on the same side also occurred, whereas the opposite side showed less cortical bone thinning (22).

Differently from most studies, we evaluated each maxillary and mandible incisor. Lund *et al*. (15) investigated the alveolar bone height on all tooth surfaces in adolescents’ extraction treatments. They observed a statistical but minimal difference between the right and left sides for all surfaces, except for the mesial surface.

We used a voxel size of 0.3 mm, which might have increased the percentage of alveolar bone loss because cortical bone thinner than 0.3 mm, although present, might not have been detected. However, Menezes *et al*. (14) demonstrated good accuracy for 0.2 mm and 0.3 mm voxel sizes to alveolar bone level measurements. In addition, Sun *et al*. (13) highlighted that this method might overestimate the actual measurements when low voxel sizes, such as 0.125, are used. Besides, a smaller voxel size did not imply a direct relationship with a greater precision of linear measurements (30). According to a previous study, the CBCT measurements proved to be reproducible in different acquisition protocols of images and different voxel(14).

## Conclusions

Orthodontic treatment with a self-ligating passive system showed a bone alveolar loss in the maxillary and mandibular central and lateral incisors.

Differences in dental inclinations were observed between the incisors.

There was no correlation between marginal alveolar buccal bone loss and incisor inclination.
